# Anti-Inflammatory Effects of Asian Fawn Lily (*Erythronium japonicum*) Extract on Lipopolysaccharide-Induced Depressive-Like Behavior in Mice

**DOI:** 10.3390/nu12123809

**Published:** 2020-12-11

**Authors:** Dong Wook Lim, Joon Park, Daeseok Han, Jaekwang Lee, Yun Tai Kim, Changho Lee

**Affiliations:** 1 Research Division of Food Functionality, Korea Food Research Institute, Wanju 55365, Korea; dwlim@kfri.re.kr (D.W.L.); 50029@kfri.re.kr (J.P.); imissu@kfri.re.kr (D.H.); jklee@kfri.re.kr (J.L.); 2 Division of Food Biotechnology, University of Science & Technology, Daejeon 34113, Korea

**Keywords:** *Erythronium japonicum*, lipopolysaccharide, anti-inflammation, depression

## Abstract

Neuroinflammation is associated with an increased risk of depression. Lipopolysaccharide (LPS) treatment is known to induce pro-inflammatory cytokine secretion and a depressive-like phenotype in mice. Although *Erythronium japonicum* exhibits various health benefits, the role of *E. japonicum* extract (EJE) in inflammation-associated depression is unknown. This study aimed to explore the anti-inflammatory effect of EJE on LPS-induced depressive symptoms in mice using the open field test (OFT), passive avoidance test (PAT), tail suspension test (TST), and forced swim test (FST). LPS-treated mice had significantly increased immobility time in the TST and FST, decreased step-through latency time in the PAT, and decreased locomotor activity in the OFT. However, administration of 100 and 300 mg/kg of EJE significantly improved these depressive-like behaviors. EJE also prevented the increase in mRNA levels of tumor necrosis factor-α (TNF-α), interleukin-1β (IL-1β), IL-6, and monocyte chemoattractant protein-1 (MCP-1), and the decrease in IL-10 levels by inhibiting nuclear factor-κB (NF-κB) subunit p65 phosphorylation. Additionally, LPS-treated mice showed markedly decreased brain-derived neurotrophic factor (BDNF) levels and phosphorylation of phosphoinositide 3-kinase (PI3K) and Akt, while EJE treatment significantly increased these levels in the hippocampus. These results suggest that EJE ameliorated LPS-induced depressive-like behavior by reducing LPS-induced neuroinflammation and activating the BDNF-PI3K/Akt pathway.

## 1. Introduction

Depression is one of the most significant mental disorders worldwide. It is estimated that more than 350 million people have depression worldwide [1], and that depression will be the most important cause of disability by 2020 [2]. Although the pathogenesis of depression remains unclear, numerous studies have reported the importance of neuroinflammation in the development of depression [3,4]. Continuous inflammation with elevated levels of pro-inflammatory cytokines can cause depressive symptoms [5]. Pro-inflammatory cytokines including interleukin-6 (IL-6) and tumor necrosis factor-α (TNF-α) levels have been found to be higher in major depressive patients [6], whereas the antioxidant capacity was lower [7]. It was reported that the cyclooxygenase-2 (COX-2) inhibitor celecoxib may be an effective adjuvant agent in the management of patients with major depression [8]. Celecoxib or ibuprofen as non-steroidal anti-inflammatory drugs (NSAIDs) may have an antidepressant effect through inhibition of inflammatory mediators such as prostaglandin E_2_ (PGE_2_) and nitric oxide (NO) in in vivo models [9,10]. Furthermore, antidepressants such as fluoxetine have anti-inflammatory properties [11,12].

As currently available antidepressants are not completely effective and have numerous undesirable side effects [13], alternative therapies including traditional herbal extracts such as St. John’s Wort or dietary supplements have been continuously used as agents to control depression [14,15]. *Erythronium japonicum* is a plant widely distributed in Eastern Asia. The aerial part of this herb is an edible wild vegetable that is traditionally used as a folk medicine in Korea. Previous studies have reported that *E. japonicum* crude extract has free radical scavenging [16], antioxidant [17], and anti-asthma [18] activities. Recently, our research group reported that *E. japonicum* extract can ameliorate inflammatory pain by suppressing pro-inflammatory cytokines in Complete Freund’s Adjuvant (CFA)-induced paw edema in mice [19]. These reports indicate that *E. japonicum* extract has potential anti-inflammatory properties. However, the anti-inflammatory effects of *E. japonicum* extract on depressive-like behavior in mice is still not well defined.

This aim of the study was to determine the anti-inflammatory effects of *E. japonicum* extract (EJE) in a depressive mouse model induced by repeated lipopolysaccharide (LPS) injection [20]. Depressive-like behaviors were evaluated using the open field test (OFT), passive avoidance test (PAT), tail suspension test (TST), and forced swim test (FST). Moreover, LPS-induced neuroinflammatory response was assessed by real-time reverse transcription polymerase chain reaction (RT-PCR) and western blot analysis.

## 2. Materials and Methods

### 2.1. Sample Preparation

**Figure 1:**
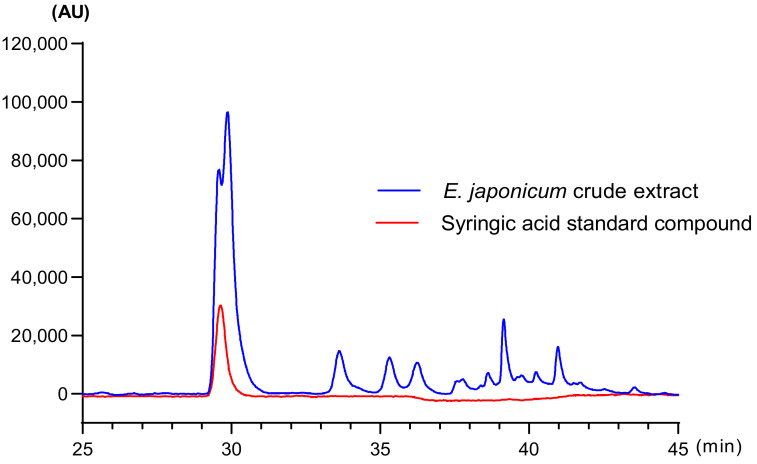
High-performance liquid chromatography (HPLC) chromatogram for standardization of the *E. japonicum* extract (EJE). The concentration of syringic acid as a standard compound was 7.6 ± 0.62 mg/g. AU, area unit.

*E. japonicum* was extracted as previously described [19]. The dried aerial parts of *E. japonicum* were purchased from Seorak Saradeul (Inje, Korea). The powdered sample (500 g) was extracted in water (10 times, *v*/*v*) using a reflux apparatus at 70 °C for 6 h. The yield of the dried extract was approximately 24.1%. The dissolved extract (50% methanol) was filtered and injected into a high-performance liquid chromatography (HPLC) system (Jasco, Hachioji, Tokyo, Japan). HPLC chromatograms were analyzed with a Waters Symmetry C18 5-μm column (4.6 × 250 mm, at 30 °C) by gradient elution, with a mobile phase composed of water, 0.2% (*v*/*v*) formic acid (A), and MeOH solution (B). The gradient was reduced by 100% (A) from 0 to 15 min, 85% (A) from 15 to 20 min, 80% (A) from 20 to 35 min, 60% (A) from 35 to 45 min, 50% (A) from 45 to 50 min, and restored by 100% (A) from 50 to 60 min. The concentration of syringic acid as a standard compound was 7.6 ± 0.62 mg/g (Figure 1).

### 2.2. Animals

Four 7-week-old male ICR mice (20–25 g, Koatech Animal Inc., Pyeongtaek, Korea) were housed four mice per cage. Mice were provided with ad libitum access to food and water under a controlled temperature (21 ± 2 °C) with a 24 h (12 h:12 h) light–dark cycle: lights on at 07:00 and lights off at 19:00 The mice were acclimated for a minimum of one week prior to beginning the study. All animal experiments were approved by the Institutional Animal Care and Use Committee (IACUC) of the Korea Food Research Institute (KFRI-M-20015).

### 2.3. Experimental Design and Sample Administration

**Figure 2:**
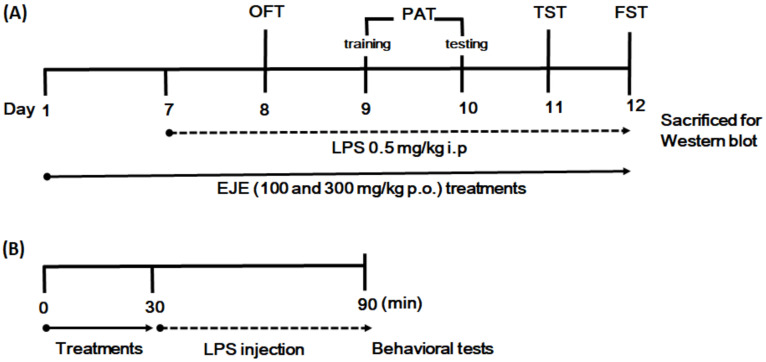
Experimental design to evaluate the effect of EJE on LPS-induced depressive mice. (**A**) After seven days of sample treatments, the mice underwent the depression-related behavioral tests. (**B**) Mice were pretreated with the samples 30 min prior to LPS injection, then, OFT, PAT, TST, and FST tests were determined 60 min after LPS injection. After the last FST session, mice were sacrificed for western blot analysis. EJE, *E. japonicum* extract; LPS, lipopolysaccharide; OFT, open field test; PAT, passive avoidance test; TST, tail suspension test; FST, forced swim test; i.p., intraperitoneal; p.o., per oral.

*E. japonicum* extract (EJE) and imipramine were dissolved in distilled water. To determine the relevant dose of EJE in the current study, we selected the dose for administration according to our previous reports [19,21,22]. EJE was treated by oral administration, while the sham and control groups were orally administered with an equal volume of distilled water. The mice were allocated to five groups of ten mice each as follows: (1) sham group (Sham), (2) control group (Con), (3) imipramine 30 mg/kg, (IMI) (4) EJE 100 mg/kg (low dosage of EJE (EJL)), and (5) EJE 300 mg/kg (high dosage of EJE (EJH)) treated group. The depressive-like behavior model was induced by repeated injection of LPS from *Escherichia coli* (055: B5, purified by phenol extraction, Sigma Aldrich, St. Louis, MO, USA), and LPS was dissolved in 0.9% (*w*/*v*) saline. LPS (0.5 mg/kg) was intraperitoneally injected to the control and EJE-treated groups, while the sham group received an equal volume of saline. To determine relevant dose of EJE in the current study, we selected the doses for in vivo according to previous our report [19]. The LPS dosage was selected based on previous reports [20,23]. After seven days of sample treatment, the mice underwent behavior experiments starting 90 min after sample treatment, as shown in the experimental scheme (Figure 2). Mice were anesthetized with 2% isoflurane, and were sacrificed for hippocampus collection. The underlying mechanism was further detected by western blot analysis. Behavioral testing was conducted between 09:00 and 14:00.

### 2.4. Open Field Test (OFT)

The OFT was performed according to the protocol previously described [24]. Briefly, mice were tested in an open field and their movements were measured. Specifically, the duration of time spent in the zone center and periphery as well as the total movement were recorded for 5 min. The mice locomotor-related activity was analyzed by SMART v3.0 software (Panlab SL, Barcelona, Spain).

### 2.5. Passive Avoidance Test (PAT)

The PAT was conducted as previously described for depression-related cognitive function [22]. The mice were tested in the GEMINI avoidance system (SD instruments, San Diego, CA, USA). In the training trial, the mice were placed in the safe section, and the door between the two sections (safe and dark sections) were opened. When the mice were allowed to enter the dark section, the door closed automatically, and the mice received an inescapable electrical foot shock (0.5 mA for 3 s). The next day, the mice were placed in the safe section again, and the step-through latency time to enter the dark section was measured.

### 2.6. Tail Suspension Test (TST)

To verify whether EJE had antidepressant-like effects, TST was performed as previously described [21]. Briefly, mice were hung by their tails using adhesive tape and attached to a hook. Immobility time was evaluated in the 4 min using an automated device (BioSeb, Chaville, France).

### 2.7. Forced Swim Test (FST)

The FST was conducted 24 h after TST, as previously described [25]. Briefly, mice were placed in a clear cylinder (height; 13 cm, diameter; 24 cm) with water (depth; 10 cm, 22–24 °C) for 6 min. The immobility and activity times were analyzed during the last 4 min by SMART v3.0 software (Panlab SL, Barcelona, Spain).

### 2.8. Western Blotting

To study the underlying effect of EJE on antidepressants, western blot was performed according to a previous study [19]. The membrane was incubated with each specific antibody (phosphor-PI3K; 4228; CST, PI3K; 4255; CST, phosphor-Akt; 9271; CST, Akt; 9272; CST, BDNF; ab108319; Abcam, phospho-p65; 3033; CST, p65; 8242; CST, and β-actin; sc-47778; Santa Cruz) overnight at 4 °C. After washing three times with TBS-T, the membrane was incubated with each secondary antibody (Mouse; A90-116P, Rabbit; A120-101P, BETHYL) in a 5% skim milk solution for 1 h at room temperature. Proteins were detected using ECL Western Blotting Substrate (32106, Thermo Scientific, Rockford, IL, USA).

### 2.9. Quantitative Reverse Transcription Polymerase Chain Reaction (RT-qPCR)

To investigate whether EJE regulated cytokine expression in hippocampus, we conducted RT-qPCR, as previously described [19]. The cytokines were detected with each primer (Table 1). Thermal cycling was carried out in a QuantStudio™ Flex Real-Time PCR system (Applied Biosystems) according to the manufacturer’s protocol. Gene expression levels were normalized to the expression levels of the β-actin housekeeping gene. Relative changes in gene expression, calculated using the 2-∆∆CT method, are reported as number-fold changes compared to those in the sham group.

### 2.10. Statistical Analysis

Data are presented as mean ± standard error of the mean (SEM) and analyzed using one-way analysis of variance (ANOVA), followed by Dunnett’s test for post-hoc comparisons using Prism 8 (GraphPad Software v8.0, Inc., San Diego, CA, USA). Statistical significance was considered at a *p*-value less than 0.05.

## 3. Results

### 3.1. Effect of EJE on the OFT

**Figure 3:**
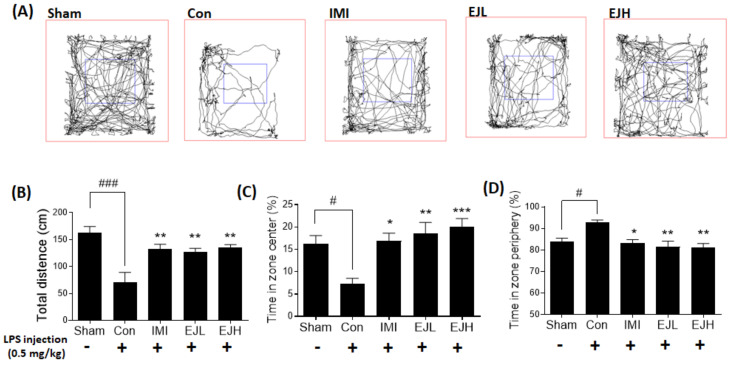
Effect of EJE on locomotor activity in LPS-induced depressive mice. The tracing of the locomotion patterns for 5 min (**A**), LPS-injected mice exhibited depressive-like behaviors, while EJ extract led to markedly improved activity; total distance (cm) (**B**), time in zone center (%) (**C**), and time in zone periphery (%) (**D**). Results are presented as mean ± standard error of the mean (SEM). EJE, *E. japonicum* extract; LPS, lipopolysaccharide; Con, control; EJL, low dosage of EJE; EJH, high dosage of EJE; IMI, imipramine 30 mg/kg; EJL, EJE 100 mg/kg; EJH, EJE 300 mg/kg. *** *p* < 0.001, ** *p* < 0.01, * *p* < 0.05 versus the LPS-injected Con; ^###^ *p* < 0.001, ^#^ *p* < 0.05, versus the Sham.

First, we examined the effect of EJE on LPS-induced abnormal behaviors in the OFT. As shown in Figure 3, LPS-injected mice exhibited depressive or anxiety-like behaviors, with a decreased total distance and time in the center zone, and increased time in the periphery zone when compared with the sham mice (Figure 3A). As expected, IMI-treated mice showed significant improvements in these LPS-induced depressive-like behaviors. EJE treatment induced significant behavioral alternations with LPS treatment, with a significant increase in the total distance (Figure 3B) (total distance (cm); Sham, 162.6 ± 11.57; Control (Con), 69.8 ± 19.19; IMI, 132.3 ± 8.58; EJL, 127.4 ± 6.52; EJH, 135.7 ± 4.89; *p*-values; F (DFn, DFd) = 1.778 (4, 20); Sham vs Con (*p* < 0.0001); Con vs IMI (*p* = 0.0033), EJL (*p* = 0.0067), and EJH (*p* = 0.0021)), and improved time in the open field center (time in the center (%); Sham, 16.3 ± 1.80; Con, 7.3 ± 1.22; IMI, 14.0 ± 3.33; EJL, 18.5 ± 2.55; EJH, 20.0 ± 1.90; *p*-values; F (DFn, DFd) = 0.3338 (4, 20); Sham vs Con (*p* = 0.0160); Con vs IMI (*p* = 0.0157), EJL (*p* = 0.0026), and EJH (*p* = 0.0007)) (Figure 3C) and periphery areas (time in the periphery (%); Sham, 83.7 ± 1.80; Con, 92.7 ± 1.22; IMI, 86.0 ± 3.33; EJL, 81.5 ± 2.55; EJH, 80.0 ± 1.95; *p*-values; F (DFn, DFd) = 0.3729 (4, 20); Sham vs Con (*p* = 0.0145); Con vs. IMI (*p* = 0.0143), EJL (*p* = 0.0025), and EJH (*p* = 0.0017)) (Figure 3D).

### 3.2. Effect on EJE on PAT

**Figure 4:**
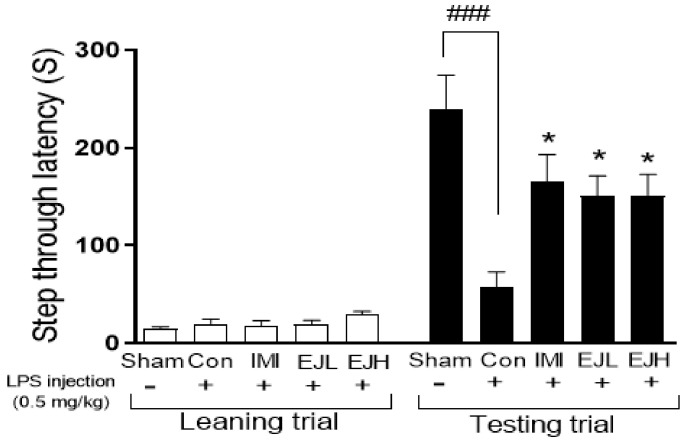
Effect of EJE on memory in LPS-induced depressive mice. EJ extract significantly improved LPS-induced memory loss in mice, indicated through an increased step through latency time (s). Results are presented as mean ± SEM. EJE, *E. japonicum* extract; LPS, lipopolysaccharide; Con, control; IMI, imipramine 30 mg/kg; EJL, EJE 100 mg/kg; EJH, EJE 300 mg/kg. * *p* < 0.05 versus the LPS-injected Con; ^###^ *p* < 0.001 versus the Sham.

To investigate the effect of EJE on LPS-induced cognitive dysfunction, one of the depressive symptoms, we performed a PAT. As expected, IMI treatment in mice recovered the LPS-induced decrease in step-through latency time. EJE treatment also significantly ameliorated the LPS-induced memory loss, indicating that EJE could alleviate depressive-like behaviors including memory deficiency in LPS-treated mice (step-through latency time (s); Sham, 240.5 ± 34.24; Con, 57.34 ± 15.98; IMI, 165.1 ± 28.21; EJL, 151.3 ± 20.11; EJH, 151.7 ± 21.17; *p*-values; F (DFn, DFd) = 0.4679 (4, 35); Sham vs Con (*p* < 0.0001); Con vs IMI (*p* = 0.045), EJL (*p* = 0.0378), and EJH (*p* = 0.0369)) (Figure 4).

### 3.3. Effect of EJE on the TST

**Figure 5:**
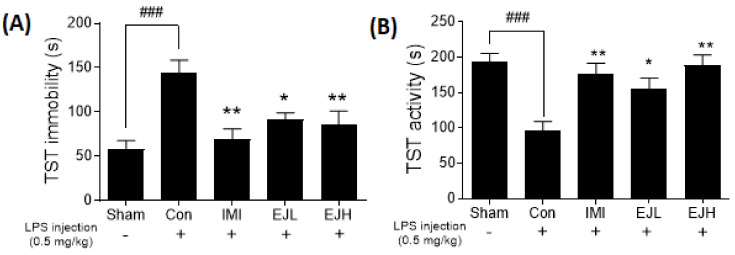
Effect of EJE on the tail suspension test in LPS-induced depressive mice. LPS-injected mice had significantly increased immobility and decreased activity, while EJ extract treated-mice showed significant improvements, with a reduced immobility time (s) (**A**) and increased activity time (s) (**B**). Results are presented as mean ± SEM. TST, tail suspension test; EJE, *E. japonicum* extract; LPS, lipopolysaccharide; Con, control; IMI, imipramine 30 mg/kg; EJL, EJE 100 mg/kg; EJH, EJE 300 mg/kg., ** *p* < 0.01, * *p* < 0.05 versus the LPS-injected Con; ^###^ *p* < 0.001 versus the Sham.

Immobility on the TST or FST is representative of a depressive-like phenotype. LPS-injected mice showed significantly increased immobility and decreased activity compared with the sham mice, while IMI treatment in mice significantly improved these depressive-like symptoms. The increased immobility caused by LPS treatment was significantly reduced by treatment with EJE (immobility time (s); Sham, 46.08 ± 13.37; Con, 144.6 ± 13.96; IMI, 71.31 ± 13.73; EJL, 91.69 ± 7.82; EJH, 85.30 ± 15.76; *p*-values; F (DFn, DFd) = 0.7969 (4, 35); Sham vs Con (*p* < 0.0001); Con vs IMI (*p* = 0.0015), EJL (*p* = 0.0497), and EJH (*p* = 0.0083)) (Figure 5A). An increase in activity was also observed in the EJE-treated group (activity time (s); Sham, 194.0 ± 13.37; Con, 95.40 ± 13.96; IMI, 168.7 ± 13.73; EJL, 154.7 ± 15.76; EJH, 148 ± 7.82; *p*-values; F (DFn, DFd) = 0.1681 (4, 35); Sham vs Con (*p* < 0.0001); Con vs IMI (*p* = 0.0054), EJL (*p* = 0.0225), and EJH (*p* = 0.0027)) (Figure 5B).

### 3.4. Effect of EJE on the FST

**Figure 6:**
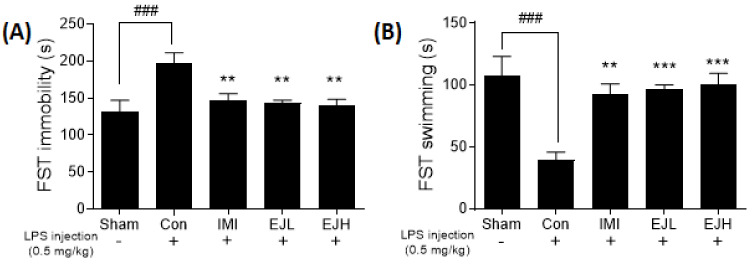
Effect of EJE on the forced swim test in LPS-induced depressive mice. LPS-injected mice showed significantly increased immobility and decreased swimming, while EJ extract-treated mice showed significant improvements, with a reduced immobility time (s) (**A**) and increased swimming time (s) (**B**). Results are presented as mean ± SEM. FST, forced swim test; EJE, *E. japonicum* extract; LPS, lipopolysaccharide; Con, control; IMI, imipramine 30 mg/kg; EJL, EJE 100 mg/kg; EJH, EJE 300 mg/kg. *** *p* < 0.001, ** *p* < 0.01, versus the LPS-injected Con; ^###^ *p* < 0.001 versus the Sham.

As shown in Figure 6, the FST results showed a similar trend to the previous TST results. The increased immobility associated with LPS was significantly reduced by the treatment with EJE (immobility time (s); Sham, 132.1 ± 15.0; Con, 212.4 ± 3.70; IMI, 149.3 ± 6.63; EJL, 150.2 ± 9.96; EJH, 138.5 ± 7.91; *p*-values; F (DFn, DFd) = 0.8466 (4, 35); Sham vs Con (*p* = 0.0006); Con vs IMI (*p* = 0.0098), EJL (*p* = 0.0051), and EJH (*p* = 0.0025)) (Figure 6A). The increasing of swimming was also observed in EJE treated group (swimming time (s); Sham, 108.0 ± 15.0; Con, 27.62 ± 3.71; IMI, 90.73 ± 6.63; EJL, 89.86 ± 9.96; EJH, 101.6 ± 7.91; *p*-values; F (DFn, DFd) = 1.516 (4, 35); Sham vs Con (*p* = 0.0003); Con vs IMI (*p* = 0.0010), EJL (*p* = 0.0005), and EJH (*p* = 0.0002)) (Figure 6B).

### 3.5. Effect of EJE on Cytokines

**Figure 7:**
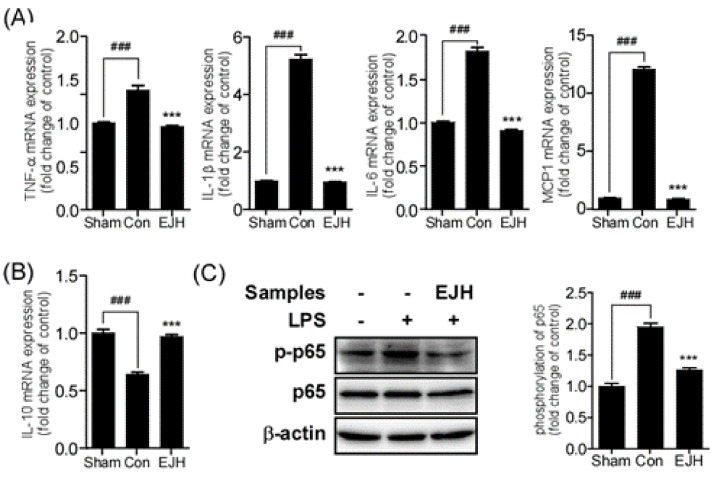
Effect of EJE on cytokines and p65 phosphorylation in the hippocampus of LPS-induced depressive mice. LPS-induced changes in cytokine mRNA expression in the hippocampus were significantly recovered by oral administration of EJH (**A**), (**B**) EJH significantly reduced phosphorylation of p65 in the hippocampus (**C**). Results are presented as mean ± SEM. EJE, *E. japonicum* extract; LPS, lipopolysaccharide; Con, control; EJH, EJE 300 mg/kg. *** *p* < 0.001 versus the LPS-injected Con; ^###^ *p* < 0.001 versus the Sham. TNF-α, tumor necrosis factor-α; IL-1β, interleukin-1β; IL-6, interleukin-6; MCP-1, monocyte chemoattractant protein-1; IL-10, interleukin-10.

Clinical and preclinical studies revealed that inflammatory cytokines were increased, while anti-inflammatory cytokines were decreased in the hippocampus of depressed patients and animals [26]. Inflammatory cytokines include TNF-α, IL-1β, IL-6, and MCP-1, and anti-inflammatory cytokines include IL-10. Recovery of these cytokines in the hippocampus might attenuate depressive symptoms. The results showed that administration of EJE significantly reduced LPS-induced mRNA levels of inflammatory cytokines (TNF-α; Sham, 1.00 ± 0.02; Con, 1.38 ± 0.11; EJH, 0.96 ± 0.03; F (DFn, DFd) = 1.397 (2, 9);/IL-1β; Sham, 1.00 ± 0.01; Con, 5.23 ± 0.3; EJH, 0.96 ± 0.02; F (DFn, DFd) = 15.62 (2, 9)/IL-6; Sham, 1.00 ± 0.02; Con, 1.82 ± 0.08; EJH, 0.91 ± 0.02; F (DFn, DFd) = 13.66 (2, 9)/MCP1; Sham, 1.00 ± 0.04; Con, 12.1 ± 0.43; EJH, 0.89 ± 0.07; F (DFn, DFd) = 5.538 (2, 9); *p*-values, Con vs. other groups (*p* < 0.0001)) (Figure 7A) and ameliorated the LPS-induced decrease in IL-10 mRNA levels (IL-10; Sham, 1.00 ± 0.06; Con, 0.64 ± 0.04; EJH, 0.97 ± 0.04; F (DFn, DFd) = 0.1473 (2, 9); *p*-values, Con vs other groups (*p* < 0.0001)) (Figure 7B) in the mouse hippocampus. NF-κB is strongly involved in regulating the expression of cytokines. To investigate the inhibitory effects on LPS-induced phosphorylation of p65, the end protein of NF-κB signaling, we performed western blotting. The results revealed that LPS-induced phosphorylation of p65 was inhibited by the administration of EJE in the hippocampus (phosphorylation of p65; Sham, 1.00 ± 0.09; Con, 1.95 ± 0.1; EJH, 1.26 ± 0.06; F (DFn, DFd) = 0.8673 (2, 6); *p*-values, Con vs other groups (*p* < 0.0001)) (Figure 7C).

### 3.6. Effect of EJE on the BDNF-PI3K/Akt Signaling Pathway

**Figure 8:**
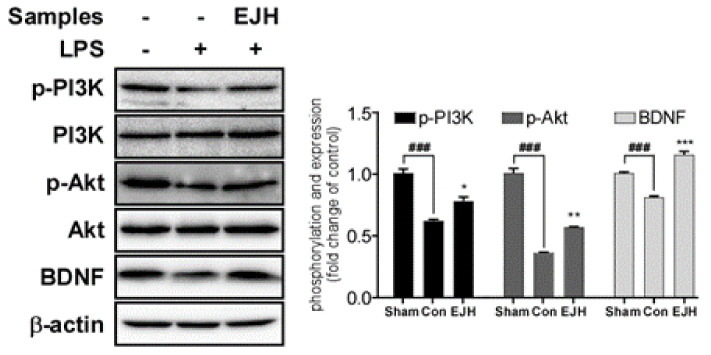
Effect of EJE on PI3K/AKT/BDNF signaling in the hippocampus of LPS-induced depressive mice. Although the PI3K/AKT/BDNF signaling was decreased by intraperitoneal injection of LPS, administration of EJH significantly increased PI3K/AKT/BDNF signaling in the hippocampus. Results are presented as mean ± SEM. EJE, *E. japonicum* extract; LPS, lipopolysaccharide; Con, control; EJH, EJE 300 mg/kg. *** *p* < 0.001, ** *p* < 0.01, and * *p* < 0.05 versus the LPS-injected Con; ^###^ *p* < 0.001 versus the Sham.

To further investigate the potential mechanism of EJE in LPS-induced depression., the BDNF-PI3K/Akt pathway in the hippocampus of LPS-treated mice was confirmed by western blotting. As shown in Figure 8, EJE treatment significantly ameliorated the LPS-induced decrease in BDNF-PI3K/Akt signaling in the (phosphorylation of PI3K; Sham, 1.00 ± 0.06; Con, 0.61 ± 0.02; EJH, 0.77 ± 0.06; F (DFn, DFd) = 0.3150 (2, 6); *p*-values; Sham vs. Con (*p* = 0.0002); Con vs. EJH (*p* = 0.0151)/phosphorylation of AKT; Sham, 1.00 ± 0.06; Con, 0.36 ± 0.01; EJH, 0.56 ± 0.01; F (DFn, DFd) = 1.132 (2, 6); *p*-values values; Sham vs. Con (*p* < 0.0001); Con vs. EJH (*p* = 0.0011)/BDNF; Sham, 1.00 ± 0.02; Con, 0.8 ± 0.02; EJH, 1.15 ± 0.05; F (DFn, DFd) = 1.007 (2, 6); *p*-values values; Sham vs. Con (*p* = 0.0006); Con vs. EJH (*p* < 0.0001)) (Figure 8).

## 4. Discussion

To the best of our knowledge, this is the first study to demonstrate that the anti-inflammatory effect of EJE significantly improves LPS-induced depressive-like behavior by activating the BDNF-PI3K/Akt pathway in the hippocampus of mice.

It is well known that macrophage-induced inflammation plays an important role in the pathophysiology of depression [27]. Activated microglia initiate an inflammatory cascade whereby the release of relevant cytokines and excessive exposure to cytokines directly affect neurons, causing cell death and decreased production of neurotransmitters or neurotrophic factors [28]. Several studies have reported increased levels of inflammatory cytokines and other mediators in patients with depression [29]. A growing body of research indicates that cytokines contribute to the development of depression [30]. Cytokines, primarily produced by glial cells, play an important role in the brain such as in development, protection, and synaptic remodeling. Cytokine expression is regulated to maintain homeostasis in the brain. However, if the microenvironment in the CNS is altered by infection, toxic stimuli, inflammation, and tissue injury, expression of pro-inflammatory cytokines increases and anti-inflammatory cytokines decreases, leading to depression [31,32]. TNF-α, IL-1β, IL-6, and MCP1 are well-known pro-inflammatory cytokines, whereas IL-10 is the most widely investigated anti-inflammatory cytokine. Previous in vivo studies have reported that inhibition of pro-inflammatory cytokines alleviated depressive symptoms [33,34,35]. Furthermore, depressive-like behaviors were attenuated by the administration of IL-10 in mice [36]. These data demonstrate that the regulation of cytokine expression can attenuate depressive symptoms. The regulation of cytokine expression is strongly involved in the NF-κB signaling pathway. In the NF-κB signaling pathway, p65 is finally phosphorylated, and the phosphorylated p65 then translocates to the nucleus to initiate transcription. The NF-κB signaling pathway is known to be associated with the pathogenesis of inflammatory diseases such as rheumatism, inflammatory bowel disease, atherosclerosis, diabetes, and pain [37,38,39]. Hence, inhibition of the NF-κB signaling pathway might be a good target to treat inflammation-related diseases such as depression. NF-κB signaling plays a critical role in the development of depression, and inhibiting NF-κB signaling can prevent depression [40,41]. In addition, many previous studies have reported that the increase in pro-inflammatory cytokines and the decrease in anti-inflammatory cytokines were attenuated by inhibition of NF-κB signaling in the hippocampus [42,43,44]. In our results, administration of EJE significantly improved LPS-induced depressive-like behavioral symptoms. Cognitive impairment or anxiety- and depression-like behaviors have been reported to occur after LPS injection [45,46,47]. It has been well known that systemic inflammatory events can induce neuroinflammation in the central nervous system [48]. Jain et al. [49] reported that systemically administered LPS increased immobility in the FST in mice. The increase in immobility in the TST or FST is reported to involve the depression behavior and can be reversed by antidepressant agents [50]. Moreover, the LPS-injected mice exhibited decreased total distance and time spent in the center area and a significant increase in total immobility time, indicating that LPS induces a number of behavioral alterations such as decreased locomotor activity [51]. The LPS-mediated cognitive dysfunction was also observed in the PAT [52]. Lee et al. [53] showed that systemic inflammation by treatment with LPS caused elevation of amyloidogenesis and neuronal cell death, which finally resulted in memory impairment. As expected, in the present study, we observed a significant reduction in locomotor and cognitive activities and increased immobility in LPS-injected mice during the OFT, PAT, TST, and FST compared to the results for the sham mice. These results are in accordance with those described in a previous report [54]. Meanwhile, EJE treatment prevented the LPS-induced depressive-like behavior in the behavioral tests. EJE also prevented the increase in mRNA levels of TNF-α, IL-1β, IL-6, and MCP-1, and the decrease in IL-10 by the inhibition of NF-κB subunit p65 phosphorylation. Therefore, our findings indicate that the LPS-induced changes in cytokines are attenuated by EJE-induced NF-κB inhibition, leading to alleviation of depression.

BDNF, a protein synthesized in the brain, has a neuroprotective function. A decreased level of BDNF in the brain, particularly the hippocampus, is a well-known symptom of depression, clinically and pre-clinically [55,56]. This is especially involved in PI3K/Akt signaling [57]. PI3K/Akt signaling is also activated by BDNF and could regulate neuronal survival and anti-apoptotic biological functions of glial cells [58]. It has been reported that PI3K/Akt signaling regulation is an important factor in the onset of depression [59]. Under depressive conditions, it was found that BDNF-PI3K/Akt signaling activity is notably reduced. Previous research has implicated that phosphorylation of PI3K/Akt signaling was reduced in the hippocampus of depressed mice [60]. In addition, depressive behavior was significantly increased in Akt-knockout mice than in wild-type mice [61]. Chen et al. [62] reported that chronic stress-induced depressive-like behaviors were inhibited by the activation of BDNF-PI3K/Akt signaling. The activation of BDNF-PI3K/Akt signaling is suggested to be therapeutically effective for depression. In our results, LPS-treated mice showed markedly decreased BDNF levels and phosphorylation of PI3K and Akt, while EJE treatment significantly increased these levels in the hippocampus. Thus, EJE attenuated depression symptoms by activating BDNF-PI3K/Akt signaling.

We found that syringic acid was contained as major components in EJE by HPLC. A growing body of evidence has shown that syringic acid had anti-inflammatory effects in various inflammation-related diseases such as obesity [63] and colitis [64]. These studies revealed that syringic acid had an anti-inflammatory effect via decreasing NF-κB activation. Indeed, brain disorders were remarkably attenuated by reducing neuroinflammation with the treatment of syringic acid in animal studies [65,66]. Oral administration of *Morus nigra* extracts, known to contain syringic acid as major components, and syringic acid significantly alleviated depressive-like behaviors in mice via regulated PI3K/Akt signaling in hippocampus [67,68]. Therefore, it might be that syringic acid is an active compound that plays a major role in EJE’s antidepressant effect. However, further studies are necessary to investigate the antidepressant-like effect of syringic acid on a LPS-induced depressive behavior model.

## 5. Conclusions

In conclusion, our findings strongly indicate that EJE ameliorated LPS-induced depressive-like behavior by activating the BDNF-PI3K/Akt pathway in the hippocampus, and this effect may be mediated by preventing LPS-induced neuroinflammation through the inhibition of NF-κB subunit p65 phosphorylation. Thus, EJE could potentially improve depressive symptoms including the inflammatory-mediated response.

## Figures and Tables

**Table 1:** Primer sequences for quantitative reverse transcription polymerase chain reaction (RT-qPCR).

Species	Gene	Primer Sequence (5′-3′)	
		Forward	Reverse
Mouse	TNF-α	CAGGCGGTGCCTATGTCTC	CGATCACCCCGAAGTTCAGTAG
	IL-1β	TTCAGGCAGGCAGTATCACTC	GAAGGTCCACGGGAAAGACAC
	IL-6	ACTCACCTCTTCAGAACGAATTG	CCATCTTTGGAAGGTTCAGGTTG
	MCP-1	CAGCCAGATGCAATCAATGCC	TGGAATCCTGAACCCACTTCT
	IL-10	GCTCTTACTGACTGGCATGAG	CGCAGCTCTAGGAGCATGTG
	β-actin	TCCGGCACTACCGAGTTATC	GATCCGGTGTAGCAGATCGC

TNF-α, tumor necrosis factor-α; IL-1β, interleukin-1β; IL-6, interleukin-6; MCP-1, monocyte chemoattractant protein-1; IL-10, interleukin-6.
